# DNA methylation of the glucagon-like peptide 1 receptor (*GLP1R*) in human pancreatic islets

**DOI:** 10.1186/1471-2350-14-76

**Published:** 2013-07-23

**Authors:** Elin Hall, Tasnim Dayeh, Clare L Kirkpatrick, Claes B Wollheim, Marloes Dekker Nitert, Charlotte Ling

**Affiliations:** 1 Department of Clinical Sciences, Lund University Diabetes Centre, CRC, Lund University, Scania University Hospital, Malmö, Sweden; 2 Department of Cell Physiology and Metabolism, University Medical Centre, 1 rue Michel-Servet, 1211, Geneva 4, Switzerland; 3 University of Queensland Centre for Clinical Research, Brisbane, Australia

**Keywords:** DNA methylation, Epigenetics, Glucagon-like peptide 1 receptor, *GLP1R*, Type 2 diabetes, Pancreatic islet, α cells, β cells, *DNMT1*, *DNMT3*

## Abstract

**Background:**

Insulin secretion is enhanced upon the binding of Glucagon-like peptide-1 (GLP-1) to its receptor (GLP1R) in pancreatic β cells. Although a reduced expression of *GLP1R* in pancreatic islets from type 2 diabetic patients and hyperglycaemic rats has been established, it is still unknown if this is caused by differential DNA methylation of GLP1R in pancreatic islets of type 2 diabetic patients.

**Methods:**

In this study, DNA methylation levels of 12 CpG sites close to the transcription start site of *GLP1R* were analysed in pancreatic islets from 55 non-diabetic and 10 type 2 diabetic human donors as well as in β and α cells isolated from human pancreatic islets. DNA methylation of *GLP1R* was related to *GLP1R* expression, HbA_1c_ levels and BMI. Moreover, mRNA expression of *MECP2*, *DNMT1*, *DNMT3A* and *DNMT3B* was analysed in pancreatic islets of the non-diabetic and type 2 diabetic donors.

**Results:**

One CpG unit, at position +199 and +205 bp from the transcription start site, showed a small increase in DNA methylation in islets from donors with type 2 diabetes compared to non-diabetic donors (0.53%, *p*=0.02). Furthermore, DNA methylation levels of one CpG site located 376 bp upstream of the transcription start site of *GLP1R* correlated negatively with *GLP1R* expression (rho=−0.34, *p*=0.008) but positively with BMI and HbA_1c_ (rho=0.30, *p*=0.02 and rho=0.30, *p*=0.03, respectively). This specific CpG site is located in an area with known SP1 and SP3 transcription factor binding sites. Moreover, when we compared the DNA methylation of the *GLP1R* promoter in isolated human β and α cells, we found that it was higher in α- compared with β-cells (*p*=0.009). Finally, there was a trend towards decreased *DNMT3A* expression (*p*=0.056) in type 2 diabetic compared with non-diabetic islets.

**Conclusions:**

Together, our study shows that while BMI and HbA_1c_ are positively associated with DNA methylation levels of *GLP1R,* its expression is negatively associated with DNA methylation of *GLP1R* in human pancreatic islets.

## Background

Glucagon-like peptide-1 (GLP-1) is an incretin hormone that is secreted by gastrointestinal L-cells in response to meal intake. The peptide is produced by the posttranslational modification of proglucagon [1]. The glucagon-like peptide-1 receptor (GLP1R) is a G protein-coupled receptor, which is expressed in the pancreas, lungs, heart, kidney, stomach, and brain [1-4]. When GLP-1 binds to its receptor in pancreatic β cells, an intracellular signalling cascade is initiated, resulting in the activation of adenylate cyclase and the formation of cAMP. The increase in cAMP enhances the secretion of insulin, providing a mechanism for GLP-1 to regulate insulin secretion in humans. Furthermore, while several studies have shown that *GLP1R* is expressed in pancreatic β cells, its expression is low or absent in pancreatic α cells [5,6].

Type 2 diabetes is a multifactorial polygenic disease characterised by chronic hyperglycaemia due to impaired insulin secretion and action. Dissecting the mechanisms that contribute to insufficient insulin secretion in type 2 diabetes patients is an important goal of understanding the disease. Disease susceptibility is affected by genetic and non-genetic factors and a combination thereof. However, epigenetic factors, including DNA methylation and histone modifications, also participate in type 2 diabetes [7]. Indeed, our group has previously demonstrated that DNA methylation correlates negatively with type 2 diabetes candidate gene expression in human pancreatic islets and skeletal muscle [7-12]. Increased DNA methylation and decreased expression of *PPARGC1A*, *INS* and *PDX1* in pancreatic islets of type 2 diabetic patients is further associated with decreased insulin secretion [8,11,13]. However, knowledge about the role of epigenetic mechanisms in the growing incidence of type 2 diabetes is still limited and additional studies analysing epigenetics in humans are hence needed.

Interestingly, *GLP1R* expression is decreased in pancreatic islets from patients with type 2 diabetes and hyperglycaemic rats [14-16]. Although the *GLP1R* promoter is GC rich and cytosine-residues in CG-dinucleotides are targets for DNA methylation, no previous study has analysed DNA methylation of the *GLP1R* promoter in human pancreatic islets. The aim of this study was therefore to analyse the levels of DNA methylation of the *GLP1R* promoter in human pancreatic islets from 55 non-diabetic organ donors and 10 donors with type 2 diabetes. DNA methylation of *GLP1R* was further correlated to gene expression, HbA_1c_ levels and BMI. We also tested if genes coding for proteins involved in epigenetic processes are differentially expressed in pancreatic islets from patients with type 2 diabetes compared with non-diabetic donors.

## Methods

### Pancreatic islets

Pancreatic islets from 55 non-diabetic and 10 type 2 diabetic deceased organ donors were obtained from the Human Tissue Laboratory at Lund University Diabetes Centre (Table 1). Islets were prepared by collagenase digestion and density gradient purification. After isolation, islets were cultured free floating in CMRL 1066 culture medium (ICN Biomedicals, Costa Mesa, CA, USA) supplemented with 10 mmol/l HEPES, 2 mmol/l l-glutamine, 50 μg/ml gentamicin, 0.25 μg/ml Fungizone (GIBCO, BRL, Gaithersburg, MD, USA), 20 μg/ml ciprofloxacin (Bayer Healthcare, Leverkusen, Germany), and 10 mmol/l nicotinamide at 37°C (5% CO_2_) prior to RNA and DNA preparation. Glucose-stimulated insulin release from the human islets was measured in duplicate during dynamic glucose perifusion (Brandel, London, UK) in order to calculate the stimulation index (SI), which was defined as the ratio between the areas under the curves that were calculated for the low (1.67 mM) and high (16.7 mM) glucose concentrations as previously described [17]. The donor before death or her/his relatives upon admission to Intensive Care Unit (ICU) had given their consent to donate organs and the local ethics committees approved the protocols.

**Table 1:** Characteristics of human pancreatic donors

**Phenotypes**	**Non-diabetic donors**	**Type 2 diabetes donors**	***p*-value**
n (male/female)	55 (29/26)	10 (6/4)	
Age (years)	56.7 ± 9.8	57.8 ± 12.6	0.74
BMI (kg/m^2^)	25.9 ± 3.6	28.1 ± 4.6	0.17
HbA_1_c	5.7 ± 0.8	7.1 ± 1.2	0.00017
Basal insulin secretion (ng/islet/h)	0.37 ± 0.27	0.22 ± 0.17	0.22
Glucose-stimulated insulin secretion (ng/islet/h)	1.42 ± 0.95	1.05 ± 1.56	0.045
Stimulation index	8.57 ± 9.62	3.07 ± 1.36	0.027

Data are expressed as mean ± SD.

### Gene expression

Total RNA was isolated with the AllPrep DNA/RNA Mini Kit (Qiagen GmbH, Hilden, Germany). RNA quality and concentration was measured using an Agilent 2100 bioanalyzer (Agilent Technologies, Inc., Santa Clara, CA, USA) and Nanodrop ND-1000 equipment (NanoDrop Technologies, Wilmington, DE), respectively. Gene expression was analysed using the Human Gene 1.0 ST Array (Affymetrix, Santa Clara, CA, USA) analysis following the Affymetrix standard protocol. The array data was summarised and normalised with Robust Multi-array Analysis (RMA) method using the software “Expression Console” (Affymetrix).

### β and α cell purification

β and α cells were purified from pancreatic islets of three human donors (aged 54, 55 and 74 years old, with BMI 21.5-23.1 kg/m^2^), different from the donors described in Table 1, using a method previously described [18,19]. In short, dissociation of islet cells was achieved by incubation with constant agitation for 3 minutes at 37°C in 0.05 % trypsin-EDTA (Life Technologies Ltd, Paisley, UK) supplemented with 3 mg/ml DNAse I (Roche, Basel, Switzerland) followed by vigorous pipetting. Labelling and FACS sorting of the β- and α-cell fractions was performed as previously described [19]. Sorted α and β-cells were applied to microscope slides and co-immunostained for insulin and glucagon in order to detect the amount of α-cells in the β-cell fraction, and vice versa. Using this method, a β-cell purity of 89 ± 9 (mean ± SD) was achieved [19].

### DNA methylation

500 ng of genomic DNA was bisulfite treated using the EZ DNA Methylation kit (Zymo Research, Orange, CA, USA). DNA methylation analysis was performed with EpiTYPER using Sequenom MassARRAY system (Sequenom, Inc., San Diego, CA, USA) as previously described [11]. Two EpiTYPER assays were designed using the online EpiDesigner tool [20], covering a total of 18 CpG sites in the region upstream or downstream of the transcription start site of the *GLP1R* gene. Primer information is given in Additional file 1: Table S1. Due to either high or low mass of the cleavage product, no data was generated for 6 CpG sites. Because of the base specific cleavage of the EpiTYPER method, two CpG sites positioned downstream of the transcription start site of the *GLP1R* gene were analysed as a unit and therefore called CpG site +199/+205.

### Statistical analysis

Statistical analyses were performed using PASW Statistics 18 for Windows (SPSS, Chicago, IL, USA). Non-parametric two samples test, Mann–Whitney U test, was performed to analyse differences between type 2 diabetes and non-diabetic donors. Correlations were analysed using the non-parametric Spearman correlation using all individuals in the study. Paired samples t-test was used to analyse the difference in methylation between α and β cells. The *p*-values presented in this study have not been corrected for multiple testing. All data is presented as mean ± sd and data in figures as mean ± SEM.

## Results

**Figure 1:**
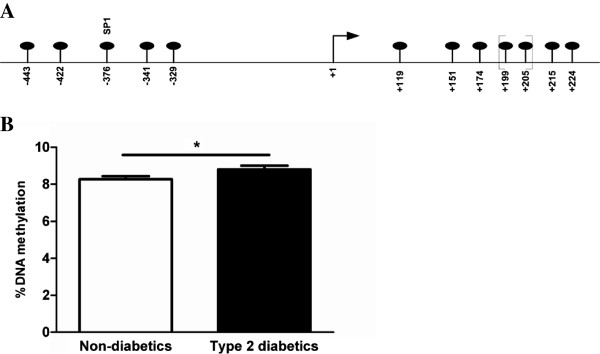
**CpG sites analysed in this study and DNA methylation difference of CpG site +199/+205. A)** Schematic figure showing the CpG sites in regions upstream and downstream of transcription start site (marked with an arrow and +1) of the *GLP1R* gene analysed in this study. The positions of the specific CpG sites are indicated in relationship to the transcription start site. One previously known transcription factor binding site that co-localises with CpG site −376 is indicated above the specific CpG site. CpG sites +199 and +205 were analysed as a unit, which is marked with brackets. **B)** The degree of DNA methylation of CpG site +199/+205 in non-diabetic and type 2 diabetic islets. Data are expressed as mean ± SEM. (* p<0.05).

**Figure 2:**
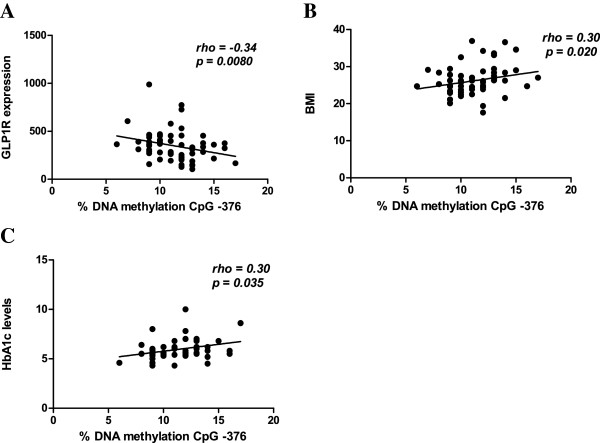
**DNA methylation of CpG site -376 correlates negatively with *GLP1R *expression and positively with BMI and HbA1c levels.** Correlation between the degree of DNA methylation of CpG site −376 of the *GLP1R* gene in pancreatic islets and **A)***GLP1R* mRNA expression, **B)** BMI and **C)** HbA_1c_ levels in all individuals of the studied cohort.

Pancreatic islets from 55 non-diabetic and 10 type 2 diabetic human donors were analysed in this study. Donor characteristics are described in Table 1. Pancreatic islets from type 2 diabetic organ donors showed a decrease in glucose-stimulated insulin secretion compared with islets from non-diabetic donors. In agreement with previous studies [14,16], we found that *GLP1R* expression was reduced in pancreatic islets from donors diagnosed with type 2 diabetes compared with non-diabetic donors (type 2 diabetic 213±76.6 vs. non-diabetic 390.4±170.2, *p*=0.0006). Furthermore, *GLP1R* mRNA expression in the human islets correlated positively with the stimulation index (SI) of glucose-stimulated insulin secretion (Table 1) (rho=0.33, *p*=0.015). We next quantified DNA methylation levels of 12 CpG sites of the *GLP1R* gene, including 5 CpG sites upstream of and 7 CpG sites downstream of the transcription start site (Figure 1A). Two of the studied CpG sites, +199 and +205 bp from the transcription start site, were analysed as a CpG unit, due to the sequence characteristic of *GLP1R*. The majority of the analysed CpG sites displayed a degree of DNA methylation below 10% (Additional file 2: Table S2). The CpG unit including CpG sites +199 and +205 showed increased DNA methylation in islets from donors with type 2 diabetes compared with non-diabetic donors (Figure 1B). However, the observed difference was small (0.53%, *p*=0.02) and did not persist after correction for multiple testing. Because previous studies have shown that increased DNA methylation may be associated with decreased gene expression [21], we next examined if DNA methylation of *GLP1R* correlates with its gene expression in the human islets. The CpG site located 376 bp upstream of the transcription start site showed negative correlation with *GLP1R* expression (rho=−0.34, *p*=0.008) (Figure 2A). In addition, the same CpG site at position −376 correlated positively with both BMI (rho=0.30, *p*=0.02) and HbA_1c_ levels (rho=0.30, *p*=0.03) (Figure 2B and C). There were no significant correlations between the degree of DNA methylation of the CpG unit including CpG sites +199 and +205 and *GLP1R* mRNA expression, BMI or HbA_1c_ levels (*p*>0.05). Neither did DNA methylation of the other analysed CpG sites correlate negatively with GLP1R mRNA expression or positively with BMI and HbA_1c_ levels (p>0.05). Transcription factor binding sites around position −376 were queried *in silico* with a web-based tool, TFsearch [22]. CpG site −376 is located in a putative SP1 transcription factor binding site as marked in Figure 1A.

**Figure 3:**
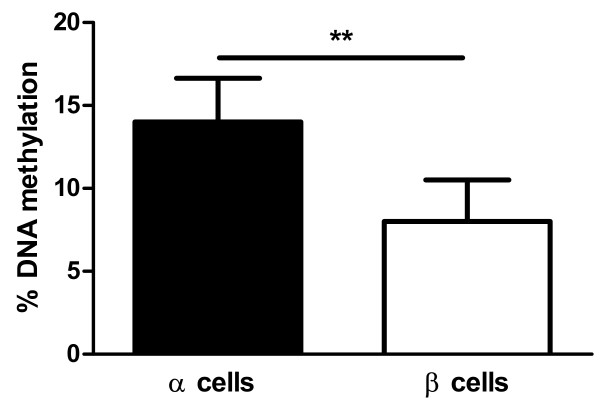
**The degree of DNA methylation of CpG site −376 of the *GLP1R *gene in α and β cells from pancreatic islets from three human donors.** Data are expressed as mean ± SEM. (** p<0.01).

Moreover, since it has been shown that *GLP1R* mainly is expressed in β cells of pancreatic islets [6], we tested if DNA methylation of the *GLP1R* promoter differed in α and β cells isolated from three human pancreatic islet donors. We found a significant increase in DNA methylation of CpG site −376 in α cells compared with β cells (α cells 14±5 vs. β cells 8±4, *p*=0.009) (Figure 3). However, there were no differences in DNA methylation between α and β cells for the other analysed CpG sites (Additional file 3: Table S3).

**Figure 4:**
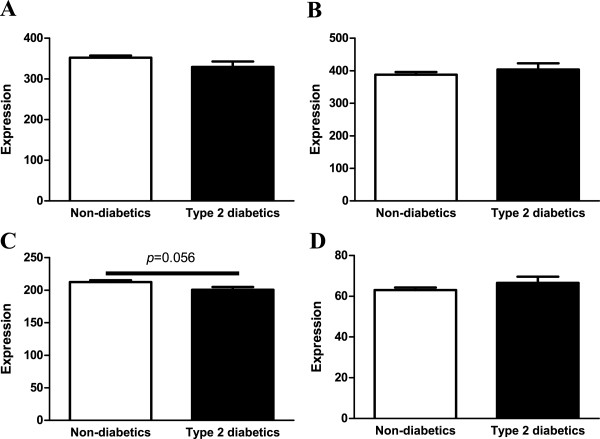
***MECP2*, *DNMT1, DNMT3A *and *DNMT3B *expression in human pancreatic islets.** Gene expression of **A)***MECP2*, **B)***DNMT1*, **C)***DNMT3A* and **D)***DNMT3B* in pancreatic islets of human non-diabetic and type 2 diabetic donors. Data are expressed as mean ± SEM.

Finally, we tested if the methyl binding protein *MECP2*, which is known to control gene expression through the interaction with transcriptional repressors [7], as well as three DNA methyl transferases; *DNMT1*, *DNMT3A* and *DNMT3B,* show differential expression in pancreatic islets from patients with type 2 diabetes. There were no differences in *MECP2*, *DNMT1* and *DNMT3B* expression between diabetic and non-diabetic donors (Figure 4A, B, D). However, there was a trend towards decreased expression of *DNMT3A* (*p*=0.056) in diabetic versus non-diabetic islets (Figure 4C).

## Discussion

A number of studies have shown differential DNA methylation of a number of type 2 diabetic candidate genes in human pancreatic islets pointing to a potential role of DNA methylation in the pathogenesis of the disease [8,11-13,23,24]. The present study suggests that DNA methylation of a CpG site located 376 bp upstream from the transcription start site in the promoter of *GLP1R* may affect the expression of this gene. We found an inverse correlation between DNA methylation of this CpG site and *GLP1R* gene expression, suggesting that methylation of this specific CpG site could have a negative effect on *GLP1R* gene expression. Wildhage et al. have reported that the *GLP1R* promoter contains at least three SP1 binding sites which SP1 can bind to. The related transcription factor SP3 can bind to only one of the sites [25]. CpG −376 is located in the binding site to which both SP1 and SP3 can bind. While SP1 in most cases serves as a transcriptional activator, SP3 has been reported to act as both an activator and as a repressor, depending on cell and tissue type. The role of SP3 in regulating gene expression depends on several different factors, including relative levels of SP1 and SP3, the number of SP1 binding sites in a promoter, cell and/or tissue type, interaction with other proteins, and chemical modification of the transcription factor. [26] Increased DNA methylation of promoter regions is associated with transcriptional silencing [21], either through preventing the binding of specific transcription factors or through recruitment of methyl CpG binding proteins, *e.g.* MeCP2, that promote recruitment of histone deacetyltransferases and/or co-repressors. DNA methylation of CpG −376 could potentially repress the binding of SP1 to the *GLP1R* promoter and hence result in transcriptional silencing. However, additional functional studies are required to examine the specific role of *GLP1R* DNA methylation on gene transcription.

It has previously been established that both protein and mRNA expression of *GLP1R* is reduced in islets from patients with type 2 diabetes compared with non-diabetic controls [14,16] as well as in rodent islets exposed to hyperglycaemia [15]. Our study identified one CpG site showing a small increase in *GLP1R* DNA methylation in islets from type 2 diabetes patients. However, the *GLP1R* promoter is highly GC rich making assay design for DNA methylation analysis in large parts of the *GLP1R* promoter inaccessible for analysis due to the requirement for CpG-free primers when using standard techniques *e.g.* EpiTYPER or Pyrosequencing. Hence, we cannot exclude that other areas of the *GLP1R* may also show differential DNA methylation in type 2 diabetes islets.

Hyperglycaemia, a high fat diet and obesity have previously been associated with differential DNA methylation in target tissues for type 2 diabetes such as pancreatic islets, skeletal muscle and liver [11,13,27-30]. For instance, HbA_1c_ has been shown to correlate positively with DNA methylation of *INS* and *PDX-1* in human pancreatic islets and hyperglycaemia was associated with increased DNA methylation of the same genes in clonal β cells cultured *in vitro*. Based on the positive correlations between both HbA_1c_ and BMI and DNA methylation of CpG site −376, the present study suggests that hyperglycaemia and/or obesity may affect DNA methylation of *GLP1R* in human islets. Interestingly, hyperglycaemia has previously been shown to increase the expression of a methyl transferase, *Dnmt1*, in clonal β cells [13]. Elevated levels of this methyl transferase may be a mechanism behind glucose induced DNA methylation. However, we did not find any significant mRNA expression differences of *DNMT1*, *DNMT3A* and *DNMT3B* in pancreatic islets from patients with type 2 diabetes. There was a trend towards a decreased expression of *DNMT3A* in type 2 diabetics compared with non-diabetics, but the difference was small and not significant (200.8±13.8 and 212.7±19.1 respectively, *p*=0.056). It is further possible that the levels of methyl donors affect the degree of DNA methylation in diabetic patients. Indeed, the level of S-adenosylmethionine, a methyl group donor, has been reported to be decreased in the erythrocytes of patients with type 2 diabetes [31].

## Conclusion

Overall, our data suggest that DNA methylation influences gene expression of *GLP1R* in human pancreatic islets. Decreased binding of the transcription factors SP1 and SP3 due to increased methylation may be involved in reducing *GLP1R* expression. Furthermore, reduced islet *GLP1R* levels associate with lower insulin secretion, which is seen in patients with type 2 diabetes. The results of this study again indicate that epigenetic mechanisms may contribute to type 2 diabetes, however additional studies are needed to fully understand the mechanisms involved in this regulation.

## Competing interests

The authors declare that they have no competing interests.

## Authors’ contributions

EH designed and conducted the study, collected, analysed and interpreted data and wrote the manuscript. TD collected and interpreted data and reviewed and edited manuscript. CLK collected data and reviewed and edited manuscript. CBW interpreted data and reviewed and edited the manuscript. MDN interpreted data and reviewed and edited the manuscript. CL designed and conducted the study, interpreted data and reviewed and edited the manuscript. EH and CL are guarantors of this work and, as such, had full access to all of the data in the study and take responsibility for the integrity of the data. All authors read and approved the final manuscript.

## Pre-publication history

The pre-publication history for this paper can be accessed here:

http://www.biomedcentral.com/1471-2350/14/76/prepub

## Supplementary Material

Additional file 1: Table S1 Primer sequence for the EpiTYPER assays. Click here for file

Additional file 2: Table S2 Degree of DNA methylation (%) for the analysed CpG sites of the *GLP1R* promoter and gene in pancreatic islets from non-diabetic and type 2 diabetic donors. Click here for file

Additional file 3: Table S3 Degree of DNA methylation (%) for the analysed CpG sites of the *GLP1R* promoter in α and β cells from 3 human pancreatic organ donors. Click here for file
