# Supplementary material for: DNA methylation of the glucagon-like peptide 1 receptor (GLP1R) in human pancreatic islets
Source: BMC Med Genet. 2013 Jul 23;14:76. doi: 10.1186/1471-2350-14-76 (PMC3727960; doi:10.1186/1471-2350-14-76)
Supplement: Additional file 1: Table S1 — Primer sequence for the EpiTYPER assays. [file 1471-2350-14-76-S1.doc]

| **Supplementary Table 1:** Primer sequence for the EpiTYPER assays. | | |
| --- | --- | --- |
| **Position** | **Direction** | **Sequence** |
| -482 -304 | Forward | aggaagagagTTGGTTTTGAGGATATTTTTTAGGA |
|  | Reverse | cagtaatacgactcactatagggagaaggctAACCCTCCTCCCACCCTAAC |
| +85 +271 | Forward | aggaagagagGTTTTTAGGTGAGATTTAGGGATTT |
|  | Reverse | cagtaatacgactcactatagggagaaggctAAACCTTCCAACCCAAAAACA |
