# Supplementary material for: DNA methylation of the glucagon-like peptide 1 receptor (GLP1R) in human pancreatic islets
Source: BMC Med Genet. 2013 Jul 23;14:76. doi: 10.1186/1471-2350-14-76 (PMC3727960; doi:10.1186/1471-2350-14-76)
Supplement: Additional file 2: Table S2 — Degree of DNA methylation (%) for the analysed CpG sites of the GLP1R promoter and gene in pancreatic islets from non-diabetic and type 2 diabetic donors. [file 1471-2350-14-76-S2.doc]

| **Supplementary Table 2:** Degree of DNA methylation (%) for the analysed CpG sites of the *GLP1R* promoter and gene in pancreatic islets from non-diabetic and type 2 diabetic donors. | | | | |
| --- | --- | --- | --- | --- |
| **Position of CpG site**  (in relationship to the transcription start site) | **Non-diabetic (%)** | **Type 2 diabetic**  **(%)** | **Difference in methylation between non-diabetic and type 2 diabetic islets (%)** | ***p*-value** |
| **-433** | 30.44 ± 3.47 | 29.90 ± 6.51 | -0.54 | 0.75 |
| **-422** | 7.65 ± 1.56 | 8.40 ± 2.17 | 0.75 | 0.35 |
| **-376** | 11.12 ± 2.35 | 12.20 ± 1.32 | 1.08 | 0.092 |
| **-341** | 0.10 ± 0.45 | 0.20 ± 0.42 | 0.10 | 0.14 |
| **-329** | 2.44 ± 0.73 | 2.50 ± 0.71 | 0.06 | 0.75 |
| **+119** | 7.85 ± 2.31 | 6.22 ± 3.53 | -1.63 | 0.19 |
| **+151** | 3.48 ± 2.99 | 2.50 ± 3.79 | -0.98 | 0.47 |
| **+174** | 3.23 ± 1.28 | 2.50 ± 1.35 | -0.73 | 0.097 |
| **+199/+205** | 8.27 ± 1.22 | 8.80 ± 0.63 | 0.53 | **0.022** |
| **+215** | 1.69 ± 1.34 | 1.20 ± 1.03 | -0.49 | 0.30 |
| **+224** | 4.81 ± 1.01 | 4.60 ± 0.70 | -0.21 | 0.35 |
| Values are shown as mean ± SD | |  |  |  |
