# Supplementary material for: DNA methylation of the glucagon-like peptide 1 receptor (GLP1R) in human pancreatic islets
Source: BMC Med Genet. 2013 Jul 23;14:76. doi: 10.1186/1471-2350-14-76 (PMC3727960; doi:10.1186/1471-2350-14-76)
Supplement: Additional file 3: Table S3 — Degree of DNA methylation (%) for the analysed CpG sites of the GLP1R promoter in α and β cells from 3 human pancreatic organ donors. [file 1471-2350-14-76-S3.doc]

**Supplementary Table 3**: Degree of DNA methylation (%) for the analysed CpG sites of the *GLP1R* promoter in α and β cells from 3 human pancreatic organ donors.

| **CpG site** | **α cells (%)** | **β cells (%)** | ***p*-value** |
| --- | --- | --- | --- |
| **-443** | 24.7 ± 8.7 | 42.7 ± 34.0 | 0.53 |
| **-422** | 10.7 ± 6.7 | 8.0 ± 3.5 | 0.29 |
| **-376** | 14.0 ± 4.6 | 8.0 ± 4.4 | 0.0091 |
| **-341** | 1.0 ± 1.7 | 0.3 ± 0.6 | 0.63 |
| **-329** | 9.0 ± 8.7 | 2.7 ± 1.5 | 0.39 |

Data are analysed using a paired t-test. Values are shown as mean ± SD
